# Effects of low-intensity pulsed ultrasound (LIPUS)-pretreated human amnion-derived mesenchymal stem cell (hAD-MSC) transplantation on primary ovarian insufficiency in rats

**DOI:** 10.1186/s13287-017-0739-3

**Published:** 2017-12-19

**Authors:** Li Ling, Xiushan Feng, Tianqin Wei, Yan Wang, Yaping Wang, Wenqian Zhang, Lianli He, Ziling Wang, Qianru Zeng, Zhengai Xiong

**Affiliations:** 1 Department of Obstetrics and Gynecology, the Second Affiliated Hospital of Chongqing Medical University, No. 76, Linjiang Road, Chongqing, 400010 China; 2 State Key Laboratory of Ultrasound Engineering in Medicine Co-Founded by Chongqing and the Ministry of Science and Technology, Chongqing Key Laboratory of Biomedical Engineering, College of Biomedical Engineering, Chongqing Medical University, Chongqing, 400010 China; 3 Department of Histology and Embryology, Laboratory of Stem Cell and Tissue Engineering, Chongqing Medical University, Chongqing, 400010 China; 4 Department of Obstetrics and Gynecology, the Third Affiliated Hospital, Zunyi Medical College, Zunyi, 563000 Guizhou China

**Keywords:** Low-intensity pulsed ultrasound (LIPUS), Human amnion-derived mesenchymal stem cells (hAD-MSCs), Primary ovarian insufficiency/failure (POI/POF), Chemotherapy, Growth factors

## Abstract

**Background:**

Human amnion-derived mesenchymal stem cells (hAD-MSCs) have the features of mesenchymal stem cells (MSCs). Low-intensity pulsed ultrasound (LIPUS) can promote the expression of various growth factors and anti-inflammatory molecules that are necessary to keep the follicle growing and to reduce granulosa cell (GC) apoptosis in the ovary. This study aims to explore the effects of LIPUS-pretreated hAD-MSC transplantation on chemotherapy-induced primary ovarian insufficiency (POI) in rats.

**Methods:**

The animals were divided into control, POI, hAD-MSC treatment, and LIPUS-pretreated hAD-MSC treatment groups. POI rat models were established by intraperitoneal injection of cyclophosphamide (CTX). The hAD-MSCs isolated from the amnion were exposed to LIPUS or sham irradiation for 5 consecutive days and injected into the tail vein of POI rats. Expression and secretion of growth factors promoted by LIPUS in hAD-MSCs were detected by real-time quantitative polymerase chain reaction (RT-qPCR) and enzyme-linked immunosorbent assay (ELISA) in vitro. Estrous cycle, serum sex hormone levels, follicle counts, ovarian pathological changes, GC apoptosis, Bcl2 and Bax expression, and pro-inflammatory cytokine levels in ovaries were examined.

**Results:**

Primary hAD-MSCs were successfully isolated from the amnion. LIPUS promoted the expression and secretion of growth factors in hAD-MSCs in vitro. Both hAD-MSC and LIPUS-pretreated hAD-MSC transplantation increased the body and reproductive organ weights, improved ovarian function, and reduced reproductive organ injuries in POI rats. Transplantation of hAD-MSCs increased the Bcl-2/Bax ratio and reduced GC apoptosis and ovarian inflammation induced by chemotherapy in ovaries. These effects could be improved by pretreatment with LIPUS on hAD-MSCs.

**Conclusion:**

Both hAD-MSC transplantation and LIPUS-pretreated hAD-MSC transplantation can repair ovarian injury and improve ovarian function in rats with chemotherapy-induced POI. LIPUS-pretreated hAD-MSC transplantation is more advantageous for reducing inflammation, improving the local microenvironment, and inhibiting GC apoptosis induced by chemotherapy in ovarian tissue of POI rats.

## Background

Primary ovarian insufficiency (POI), also known as premature ovarian failure (POF), is pathologically characterized by oligomenorrhea or amenorrhea, low levels of gonadal hormones, and high levels of follicle-stimulating hormone (FSH; > 25 mIU/ml) in females before the age of 40 years [1–3]. POI induces multiple health risks, and affects about 1% of women under the age of 40 years [2]. Chemotherapy is a commonly used treatment method for women bearing tumors, which could induce ovarian failure, including follicle loss, vascular damage, and tissue fibrosis, especially in young females [4–7]. Therefore, it is of great importance to develop and/or improve the treatment strategies for the irreversible pathogenesis of POI.

Regenerative medicine research suggests that mesenchymal stem cell (MSC) transplantation may restore the ovarian structure and function in animal models of POI, providing an effective treatment method [8, 9]. Recently, human amnion-derived mesenchymal stem cells (hAD-MSCs) have been shown to have the features of MSCs [10–12]. Self-renewal capacity, multipotency, low immunogenicity, and noninvasive application without controversy make hAD-MSCs a promising and useful source of stem cells for transplantation and regenerative medicine [11–13]. Some studies have demonstrated the efficacy of hAD-MSC transplantation for disease treatment in animal models [14–16]. However, whether hAD-MSC transplantation can restore the ovarian function in chemotherapy-induced POI is still unknown.

Low-intensity pulsed ultrasound (LIPUS) was defined as a safe and effective therapy for promoting fracture healing by the Food and Drug Administration (FDA) in 1994. However, there is currently no standard for LIPUS treatment, and studies have been conducted with intensity levels between 5 and 500 mW/cm^2^, frequencies between 45 kHz and 3 MHz, pulse repetition rate from 100 Hz to 1 kHz, and duty cycles from 20% to 50% [17–19]. It has been reported that mechanical stimulation by LIPUS is able to induce a series of biochemical events at the cellular level that can promote the secretion of various growth factors and anti-inflammatory molecules [17, 20–22], including fibroblast growth factor (FGF)2, insulin-like growth factor (IGF)-1, and vascular endothelial growth factor (VEGF). MSCs have been shown to have the ability to sense and respond to physical stimuli [23, 24]. However, whether LIPUS can promote the expression and secretion of those growth factors in hAD-MSCs are still unknown.

Apoptosis of oocyte and granulosa cells (GCs) has been identified as one possible underlying mechanism for chemotherapy-induced POI, which leads to follicular atresia and loss of ovarian reserve [25]. It has been demonstrated that, although the follicle growth is mainly regulated by pituitary gonadotropins in monovular species, mediation of some local growth factors, such as FGF2, IGF-1, hepatocyte growth factor (HGF), and VEGF, are essential to keep the follicle growing [26, 27]. Importantly, those growth factors could regulate GCs and reduce GC apoptosis [26, 28, 29]. In this study, whether LIPUS could promote the expression and secretion of those growth factors in hAD-MSCs, and whether LIPUS-pretreated hAD-MSC transplantation could be more efficient to treat patients with POI, was investigated. The efficacy of hAD-MSCs and LIPUS-pretreated hAD-MSC transplantation on chemotherapy-induced POI was evaluated in rat models.

## Methods

### Isolation and culture of hAD-MSCs

The research was in compliance with the Helsinki Declaration and approved by the Ethics Committee of the Second Affiliated Hospital of Chongqing Medical University. Primary hAD-MSCs were isolated from term amnion, according to the protocol described by Fang et al. [30]. Term placentas were collected from healthy donors who received cesarean section at the Second Affiliated Hospital of Chongqing Medical University, Chongqing, China. Written informed consent was obtained from all these donors before collection.

Amnion was separated from placenta and digested with 0.05% trypsin and 0.02% ethylenediaminetetraacetic acid (EDTA; Gibco, Grand Island, NY, USA) at 37 °C for 40 min. The mixture was filtered through a 300-mesh sieve and residues were collected (the process was repeated once). The residues were further digested with 0.75 mg/mL collagenase II (Sigma-Aldrich, St. Louis, MO, USA) and 0.075 mg/mL DNase I (Worthington, New Jersey, USA) at 37 °C for 1.5 h. After filtration and centrifugation, the isolated hAD-MSCs were cultured in the L-DMEM medium (Gibco, Grand Island, NY, USA), supplemented with 10% fetal bovine serum (FBS; Invitrogen, Carlsbad, CA, USA), 100 U/mL penicillin, and 0.1 mg/mL streptomycin (Beyotime, Haimen, Jiangsu, China), in a humidified atmosphere with 5% CO_2_ at 37 °C. Cells were subcultured at a ratio of 1:2 when 90–100% confluency was reached. The third to fifth passages (P3–5) of hAD-MSCs were used for the following experiments.

### Identification and characterization of hAD-MSCs

Primary hAD-MSCs were identified and characterized by flow cytometry. In total, 1 × 10^6^ cells were incubated with fluorescein isothiocyanate (FITC)-labeled anti-human antibodies FITC-CD105, FITC-CD45, FITC-CD34, FITC-CD19, FITC-CD14, FITC-HLA-DR, and FITC-CD90, phycoerythrin (PE)-labeled PE-CD73, or FITC- or PE-conjugated isotype control (all from BD Biosciences, San Diego, CA, USA), and analyzed on a FACSCalibur flow cytometer (BD Biosciences). Data were processed using the Cell Quest software (BD Biosciences).

To identify the multipotent differentiation of hAD-MSCs, the cells were cultured in osteogenic differentiation medium, adipogenic differentiation medium, and chondrogenic differentiation medium, respectively, for 21 days. After staining with Alizarin Red S, Oil Red O, and Alician blue (all from Cyagen Biosciences Inc., Suzhou, Jiangsu, China), respectively, the cells were observed under an inverted microscope (Olympus Corporation, Tokyo, Japan).

### LIPUS protocol

The LIPUS exposure device (Chongqing Haifu Medical Technology Co. Ltd., Chongqing, China) consisted of six transducers (34.8 mm in diameter) in an array, which was specifically designed for the six-well culture plate. LIPUS parameters were set as follows: frequency, 0.25 MHz; burst width sine wave, 200 μs; duty cycle, 20%; pulse repetition frequency, 1 kHz; spatial-average temporal-average intensity (I_SATA_), 30 mW/cm^2^; and exposure time, 30 min. The conditions have previously been proven to benefit the viability of hAD-MSCs according to our preliminary experiments (data not shown).

### Labeling and tracking of hAD-MSCs

To track and locate the transplanted hAD-MSCs in the ovarian tissues, the cells were pre-labeled with the PKH26 Red Fluorescent Cell Linker Kits (Sigma-Aldrich, St. Louis, MO, USA) [31, 32]. The PKH26-labeled hAD-MSCs were transplanted into rats via the tail vein. At 24 h, 4 weeks, and 8 weeks after cell transplantation, ovaries were fixed with optimal cutting temperature (OCT) compound and made into fresh sections (10 μm thick). After fixation with pre-cooled acetone for 10 min, the sections were washed and incubated with 2-(4-amidinophenyl)-6-indolecarbamidine dihydrochloride (DAPI; Boster Biological Technology Co. Ltd., Wuhan, Hubei, China) at room temperature for 10 min. The sections were then imaged under a fluorescent microscope (Nikon Corporation, Tokyo, Japan).

### CCK-8 assay

The cell counting kit-8 (CCK-8) assay (Beyotime) was used to detect the growth of hAD-MSCs according to the manufacturer’s instructions. Cells were seeded onto the 96-well plate at a density of 5 × 10^3^ cells/well and cultured in the incubator. The optical density (OD) value at 450 nm was then measured every day for 4 continuous days using a plate reader (1510 model; Thermo Fisher Scientific Oy, Vantaa, Finland).

### Animal model establishment and grouping

In total, 160 female Sprague-Dawley (SD) rats (10–12 weeks of age) were purchased from the Experimental Animal Center of Chongqing Medical University (Chongqing, China). Animal experimental protocols were approved by the Ethics Committee of the Second Affiliated Hospital of Chongqing Medical University. These animals were randomly divided into control, POI, hAD-MSC treatment (hAD-MSCs), and LIPUS-pretreated hAD-MSC treatment (LIPUS + hAD-MSCs) groups (*n* = 40 each group). To establish the POI model, the rats from the POI, hAD-MSCs, and LIPUS + hAD-MSCs groups received intraperitoneal injection of cyclophosphamide (CTX; Hengrui medicine Co. Ltd., Lianyugang, Jiangsu, China; re-suspended in normal saline), at a dose of 50 mg/kg on the first day and then a daily dose of 8 mg/kg for the next 14 consecutive days [33]. The rats from the control group were only injected with normal saline. At 24 h after chemotherapy, the rats from the hAD-MSCs and LIPUS + hAD-MSCs groups were injected with 0.6 mL phosphate-buffered saline (PBS) containing 4 × 10^6^ hAD-MSCs and LUPUS-pretreated hAD-MSCs labeled with PKH-26, respectively [31, 34, 35], via the tail vein [36].

At 1 week after CTX injection, the vaginal smears of ten rats from each group were obtained at 9.00 am daily for 10 weeks to observe the estrous cycle. The rats in the stages of estrus, proestrus, metestrus, and diestrus were identified and quantified by hematoxylin and eosin (H&E) staining.

From the first day of chemotherapy, animals in the four groups were weighed every week for 12 consecutive weeks. From 1 to 10 weeks after transplantation rats were narcotized and the organs and blood were collected. Organs were fixed with 4% paraformaldehyde or OCT compound (Sakura Finetek USA Inc., Torrance, CA, USA) for further processing. Serum was collected by centrifugation and stored at −80 °C for hormone tests.

### Real-time quantitative polymerase chain reaction (RT-qPCR)

The hAD-MSCs were seeded onto the six-well plate at a density of 1 × 10^5^ cells/mL. After 12 h the cells were exposed to sham irradiation (ultrasound generator was not switched on) or LIPUS for 5 consecutive days. The mRNA expression levels of growth factors (FGF2, IGF-1, HGF, and VEGF) were then detected by RT-qPCR.

Total RNA was extracted using TRIzol Reagent (Invitrogen, Carlsbad, CA, USA). The RNA sample was quantified and reverse transcribed into cDNA using the ReverTra Ace-α-first strand cDNA Synthesis Kit (TOYOBO Life Science, Shanghai, China). RT-qPCR was performed using a CFX96 Real-Time PCR Detection System (Bio-Rad, Hercules, CA, USA) with SYBR Green Real-Time PCR Master Mix (TOYOBO Life Science). Primers used in the experiment are listed in Table 1. Target gene expression was determined using the 2^−ΔΔCt^ method. GAPDH was used as the internal reference.

**Table 1:** Primer sequences for real-time quantitative polymerase chain reaction

	Primers sequence 5′–3′	Amplification	Accession no.
*FGF2*	TACCCATACAGCAGCAGCCTAGC	145 bp	NM_002006
	GCCGCCTAAAGCCATATTCATTCAC		
*HGF*	GGCCAAGTCCCCAAACAATTC	107 bp	NM_000601
	GCCGCCCTATATTCTGTGGACTAAG		
*IGF1*	CCACCACCTTTCAACTTTTTATCAC	197 bp	NM_000618
	CGCGTTTATTCTCCATGCTCTTG		
*VEGF*	GCGCAAGAAATCCCGGTATAAG	233 bp	NM_001025366
	GCCTCGGCTTGTCACATCTG		
*GAPDH*	CTCCTCCGGGTGATGCTTTTCCTAG	248 bp	NM_001256799
	CTCGCTCCTGGAAGATGGTGATG		

### TUNEL assay

Ovarian cell apoptosis was detected by the TUNEL apoptosis assay kit (Roche Applied Science, Basel, Switzerland) according to the manufacturer’s instructions. Nuclei of ovarian apoptotic cells were stained dark brown. Sections were observed and imaged by an optical microscope (Olympus Corporation, Tokyo, Japan).

### H&E staining

To analyze the ovarian morphology and follicle counts, ten ovaries from each group were collected at 1 month after hAD-MSC transplantation and fixed with 4% paraformaldehyde for 48 h. The ovaries were dehydrated, embedded in paraffin, and cut into 5-μm sections. After H&E staining, sections were observed and imaged by an optical microscope (Olympus Corporation). Follicles were counted and categorized in every fifth section through the ovary [8], which were classified as primordial, primary, secondary, preovulatory, and atretic follicles, respectively [37, 38].

### Enzyme-linked immunosorbent assay (ELISA)

Serum levels of anti-Müllerian hormone (AMH), E2, and FSH at the indicated time points (0, 2, 4, 6, 8, and 10 weeks after cell transplantation) were detected by ELISA kits (Uscn Life Science, Wuhan, Hubei, China) according to the manufacturer’s instructions.

To detect the inflammatory cytokines in the ovaries at 24 h after cell transplantation, ovarian tissue was collected from the four groups and homogenized. Ovarian supernatant was collected, and the levels of interleukin (IL)-1β, IL-6, tumor necrosis factor (TNF)-α, and VEGF were detected by ELISA kits (all from Boster Biological Technology Co., Ltd.) according to the manufacturer’s instructions.

To detect the growth factors secreted by hAD-MSCs, cells were treated as described in the RT-qPCR subsection, except for that the complete medium was replaced with serum-free medium on the third day before sham irradiation or LIPUS treatment. After 72 h, the cell supernatant was collected to detect the levels of IGF-1, HGF, and VEGF (all from Uscn Life Science) according to the manufacturer’s instructions.

### Western blot analysis

At 1 week after cell transplantation, ovaries were collected from the four groups and homogenized with RIPA lysis buffer (Beyotime). Protein concentration was determined by the Bradford Protein Assay Kit (Beyotime). Protein samples were separated by SDS-PAGE and subsequently electrically transferred onto the PVDF membrane (Millipore, Billerica, MA, USA). After washing and blocking with 5% skimmed milk for 1 h, the membrane was incubated with rabbit anti-rat primary antibodies for Bcl-2 and Bax (Abcam, Cambridge, UK) at 4 °C overnight. After washing three times, the membrane was incubated with the secondary antibodies (Cell Signaling Technology, Boston, MA, USA) at room temperature for 1 h. The BeyoECL Plus kit was used for color development according to the manufacturer’s instructions.

### Statistical analysis

Data are expressed as mean ± SD. Statistical analysis was performed with the SPSS 22.0 software (IBM, Armonk, NY, USA). Independent-samples *t* test and one-way analysis of variance (ANOVA) were used for two- and multiple-group comparisons, respectively. Statistical significance was set at *P* < 0.05.

## Results

### Identification and characterization of hAD-MSCs

**Fig. 1:**
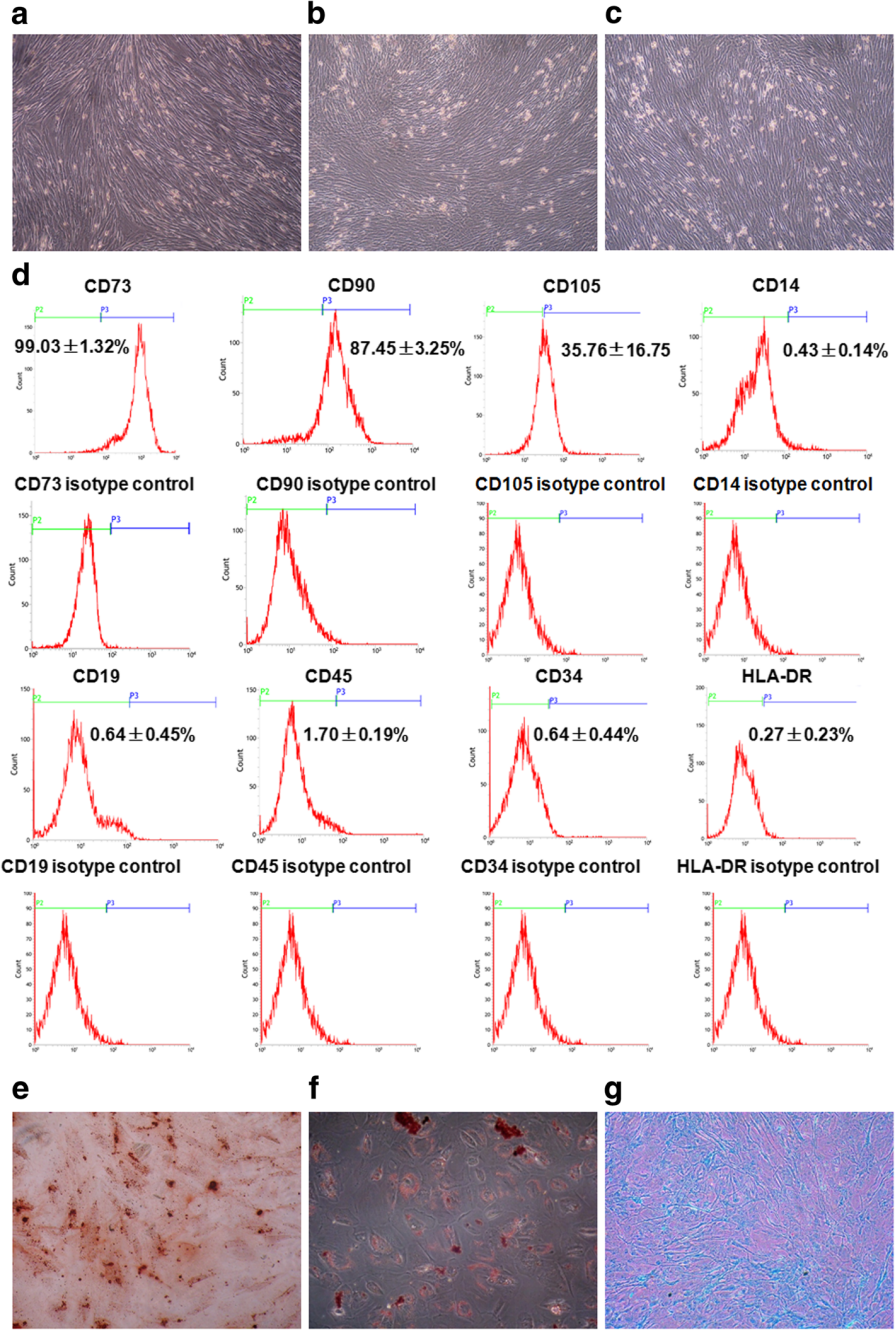
Morphology, phenotype, and multipotency of hAD-MSCs. **a**–**c** The cell morphology of passages 0 (**a**), 3 (**b**), and 5 (**c**) hAD-MSCs (100×). **d** Flow cytometry analysis of cell phenotype. **e**–**g** The osteogenic (**e**; 100×), adipogenic (**f**; 200×), or chondrogenic (**g**; 100×) differentiation of hAD-MSCs was assessed by specific staining

In our experiments, an average of approximately 3.08 ± 1.36 × 10^7^ hAD-MSCs could be yielded from each amnion. These hAD-MSCs displayed fibroblastic morphology at 10–12 days after isolation (Fig. 1a), and the subcultured cells also maintained fibroblastic morphology (Fig. 1b and c). The phenotype of hAD-MSCs (P3) was detected by flow cytometry. As shown in Fig. 1d, the phenotype characteristics of isolated hAD-MSCs were similar to bone marrow-derived MSCs (BM-MSCs). Moreover, the multipotency of hAD-MSCs was confirmed by their ability to differentiate into osteoblasts, adipocytes, and chondroblasts, respectively (Fig. 1e–g). These results indicate that the isolated cells are identified as hAD-MSCs, and have the common characteristics of multipotent MSCs.

### LIPUS promotes expression and secretion of growth factors in hAD-MSCs

**Fig. 2:**
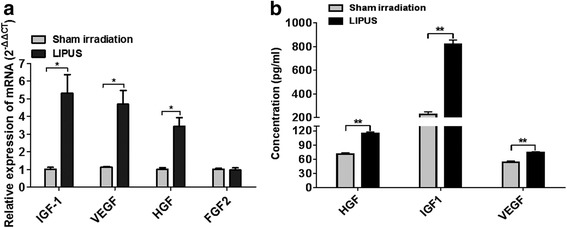
LIPUS promotes growth factor expression in hAD-MSCs. **a** The relative mRNA expression levels of FGF2, IGF-1, HGF, and VEGF in hAD-MSCs in the LIPUS and sham irradiation groups were detected by RT-qPCR. **b** The levels of IGF-1, HGF, and VEGF secreted by hAD-MSCs in the cell supernatant in the LIPUS and sham irradiation groups were detected by ELISA. *P < 0.05, **P < 0.01. *HGF* hepatocyte growth factor, *IGF1* insulin-like growth factor-1, *LIPUS* low-intensity pulsed ultrasound, *VEGF* vascular endothelial growth factor

To determine whether LIPUS could promote the expression and secretion of FGF2, IGF-1, HGF, and VEGF in hAD-MSCs, RT-qPCR and ELISA were performed. We found that the relative mRNA expression and protein secretion levels of IGF-1, HGF, and VEGF in hAD-MSCs in the LIPUS group were significantly higher than in the sham irradiation group (*P* < 0.05 and *P* < 0.01, respectively; Fig. 2b). These results demonstrate that LIPUS can promote the expression and secretion of IGF-1, HGF, and VEGF in hAD-MSCs.

### In vivo tracking of hAD-MSCs

**Fig. 3:**
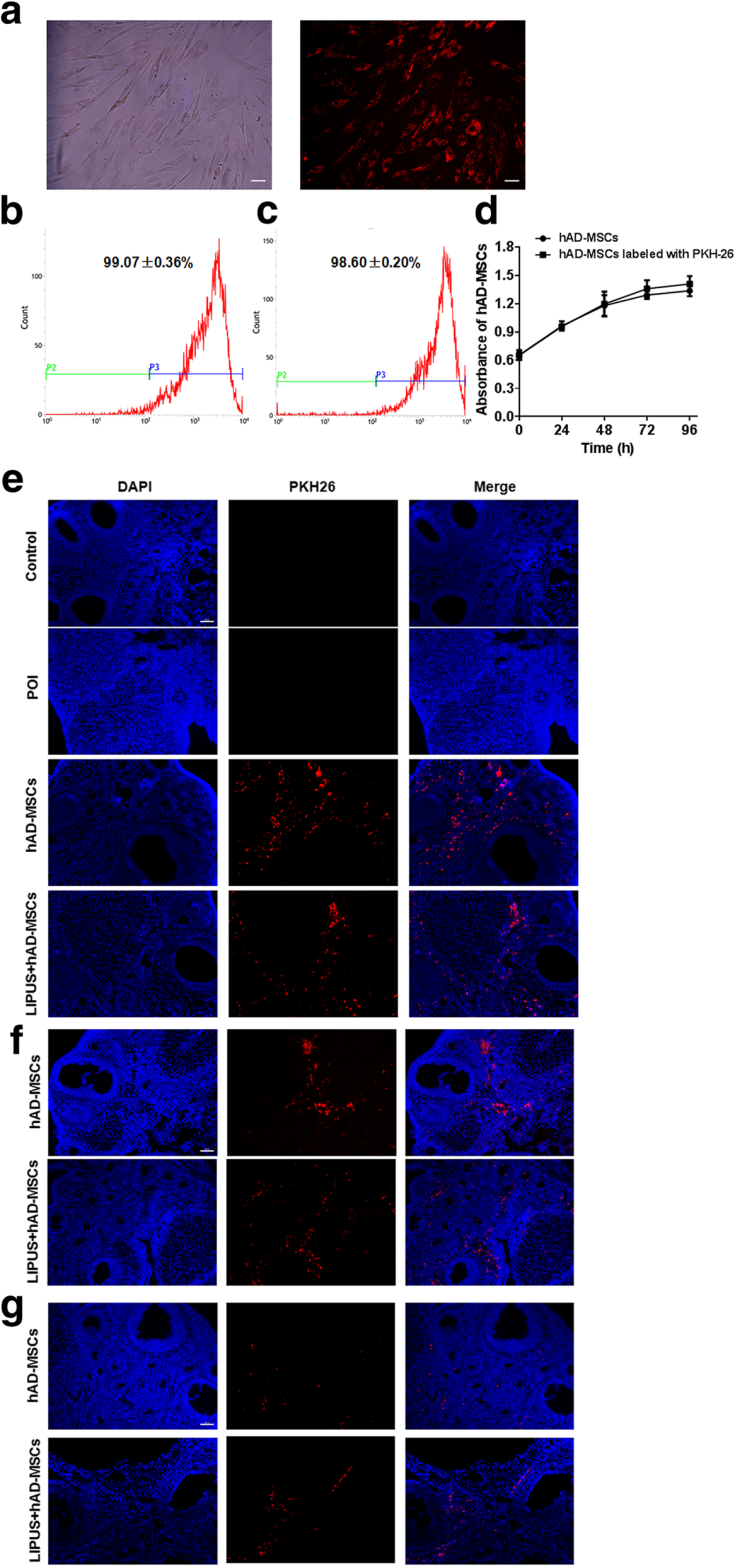
In vivo hAD-MSC tracking. **a** PKH26-labeled hAD-MSCs showed red fluorescence (100×). **b,c** The labeling rates of PKH26-labeled hAD-MSCs (**b**) and their subcultured cells (**c**) were detected by flow cytometry. **d** The growth curves of PKH26-labeled and unlabeled hAD-MSCs were measured by CCK-8 assay (*n* = 6). **e–g** Transplanted hAD-MSCs were observed at 24 h (**e**), 4 weeks (**f**), and 8 weeks (**g**) after cell transplantation in ovaries (100×). *Scale bars* = 100 μm. *P < 0.05, **P < 0.01. *hAD-MSCs* human adipose-derived mesenchymal stem cells, *LIPUS* low-intensity pulsed ultrasound, *POI* primary ovarian insufficiency

In order to track and locate the hAD-MSCs in vivo, the cells were pre-labeled with PKH26 before transplantation (Fig. 3a). As detected by flow cytometry, the cell labeling rate was 99.07 ± 0.36% (Fig. 3b), which did not decrease after cell passaging (98.60 ± 0.20%; Fig. 3c). Cell growth was investigated by the CCK-8 assay. The results showed that there was no significant change in cell activity and proliferation between PKH26-labeled and unlabeled hAD-MSCs (Fig. 3d). These results demonstrate that PKH26 labeling is efficient and stable and does not influence the activity of hAD-MSCs. The location and fate of transplanted PKH26-labeled hAD-MSCs in ovarian tissue were traced at 24 h, 4 weeks, and 8 weeks after cell transplantation (Fig. 3e–g). The results show that PKH26-labeled cells were only located in the interstitium of ovaries, rather than in follicles, after transplantation in both the hAD-MSCs and LIPUS + hAD-MSCs groups. Moreover, the red fluorescent signal could still be clearly observed in ovaries at 8 weeks after cell transplantation in those two groups.

### Transplantation of hAD-MSCs increases body and reproductive organ weights of POI rats

**Fig. 4:**
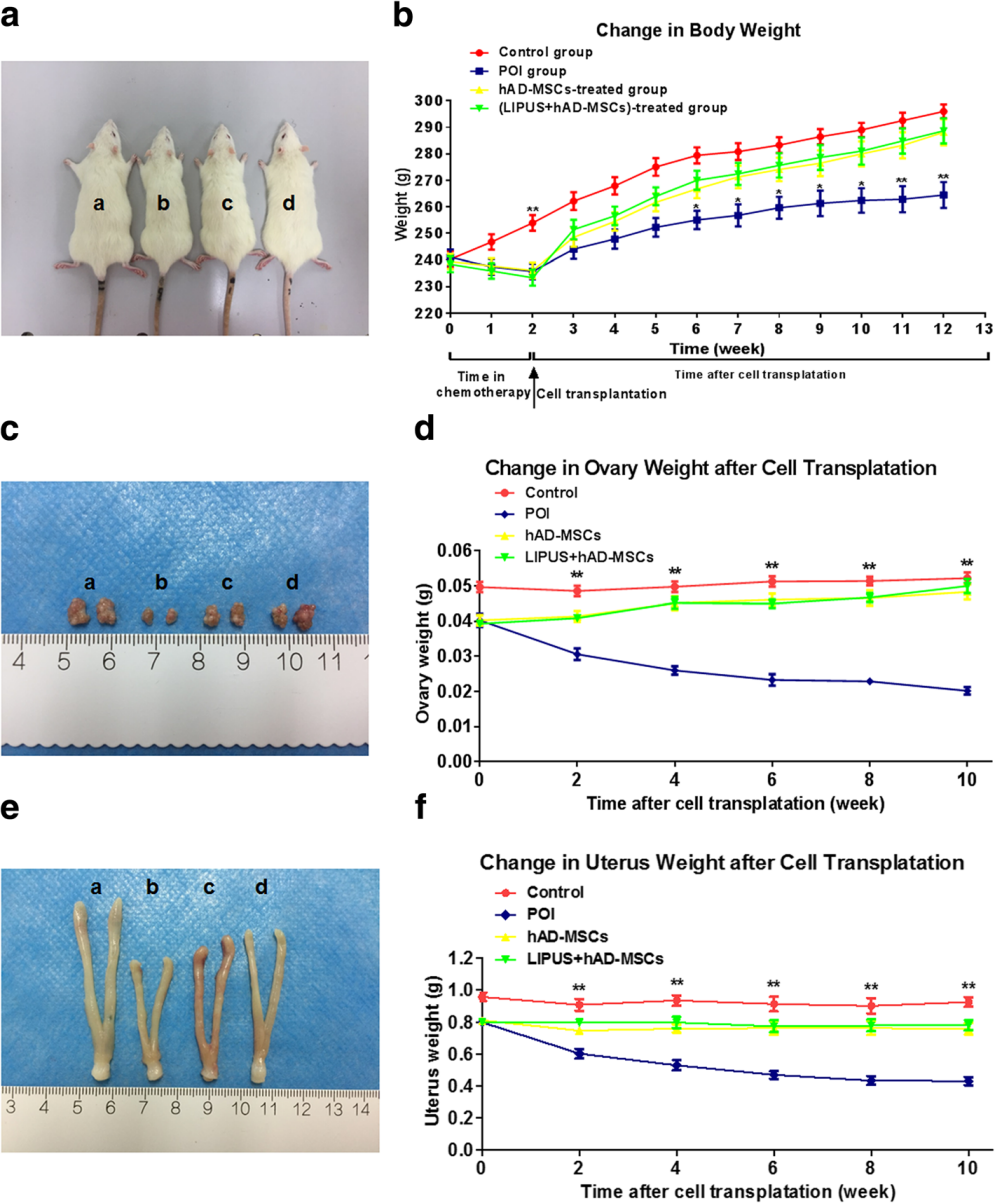
Transplantation of hAD-MSCs increases body and reproductive organ weights of POI rats. **a,b** Representative photographs of rats at 4 weeks after cell transplantation (**a**), and the changes in body weight before and after cell transplantation (**b**). **c,d** Representative photographs of ovaries at 4 weeks after cell transplantation (**c**), and the changes in ovary weight after cell transplantation (**d**). **e,f** Representative photographs of uteruses at 4 weeks after cell transplantation (**e**) and the changes in uterus weight after cell transplantation (**f**). *a* the control group, *b* the primary ovarian insufficiency (*POI*) group, *c* the human adipose-derived mesenchymal stem cells (*hAD-MSCs*) group, *d* the low-intensity pulsed ultrasound (*LIPUS*) + hAD-MSCs group. *P < 0.05, **P < 0.01

The body and reproductive organ weights of the rats were investigated next. Our results show that, compared to the control group, the body weights of rats in the POI, hAD-MSCs, and LIPUS + hAD-MSCs groups were significantly decreased after chemotherapy (*P* < 0.05; Fig. 4b). Moreover, compared to the POI group, the body weights in both the hAD-MSCs and LIPUS + hAD-MSCs groups were significantly increased, starting from the fourth week after cell transplantation (*P* < 0.05; Fig. 4a and b). However, there was no significant difference between the hAD-MSCs and LIPUS + hAD-MSCs groups. On the other hand, ovaries and uteruses were weighed at 0, 2, 4, 6, 8, and 10 weeks after cell transplantation. Our results show that, compared to the POI group, the weights of ovaries and uteruses in the hAD-MSCs and LIPUS + hAD-MSCs groups were significantly increased, starting from the second week after cell transplantation (*P* < 0.05; Fig. 4c–f). However, there was no significant difference between the hAD-MSCs and LIPUS + hAD-MSCs groups. These results demonstrate that both hAD-MSC and LIPUS-pretreated hAD-MSC transplantation can increase the body and reproductive organ weights of POI rats.

### Transplantation of hAD-MSCs improves ovarian function in POI rats

**Fig. 5:**
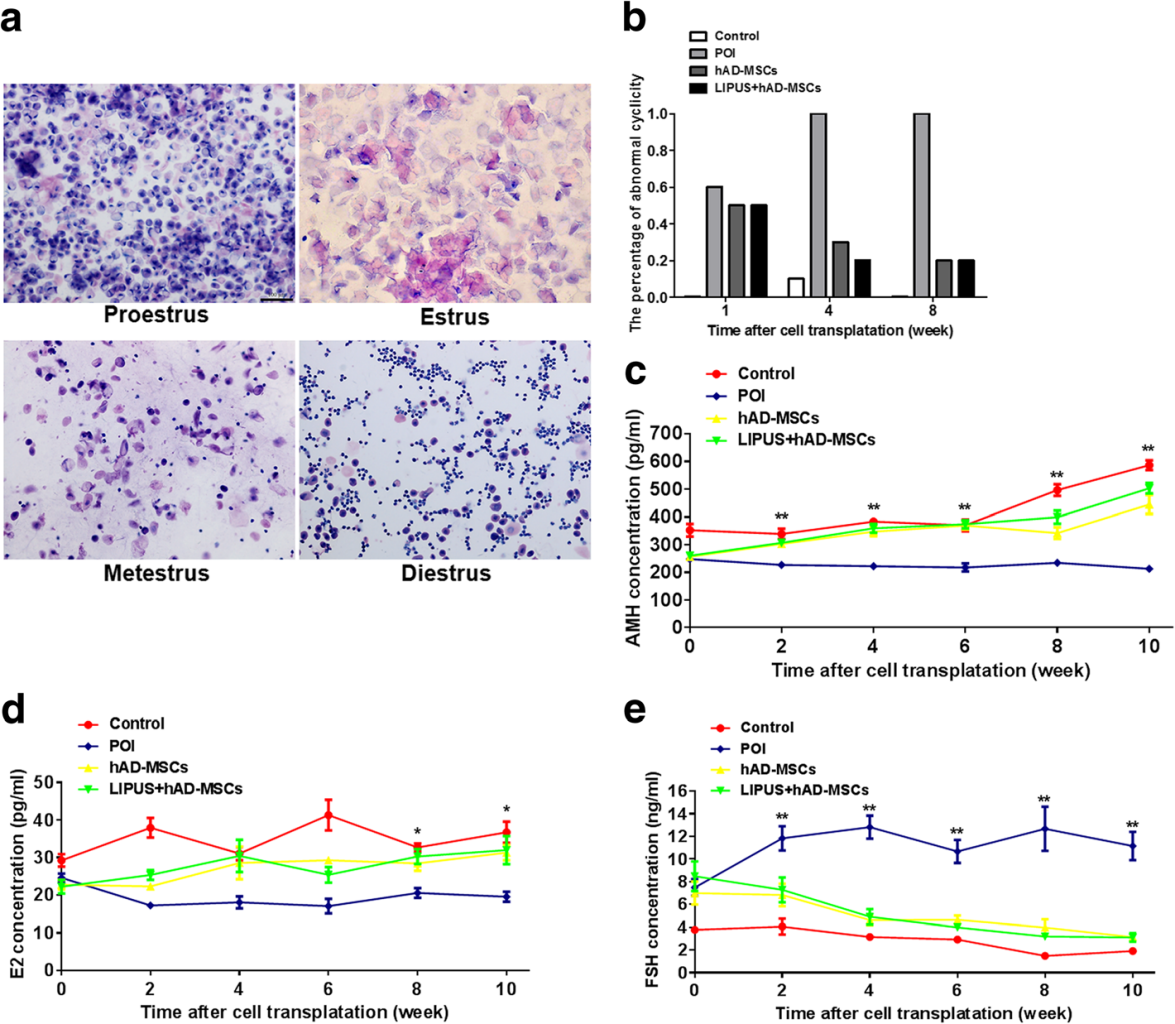
Transplantation of hAD-MSCs improves ovarian function in POI rats. **a** Vaginal smears were obtained and the estrous cycles were evaluated by H&E staining. Representative photographs for estrus, proestrus, metestrus, and diestrus are shown (100×). **b** The percentage of rats with abnormal cyclicity was detected at 1, 4, and 8 weeks after cell transplantation. **c**–**e** Serum levels of AMH (**c**), E2 (**d**), and FSH (**e**) were detected at 0, 2, 4, 6, 8, and 10 weeks after cell transplantation. *Scale bars* = 100 μm. *P < 0.05, **P < 0.01. *AMH* anti-Müllerian hormone, *FSH* follicle-stimulating hormone, *hAD-MSCs* human adipose-derived mesenchymal stem cells, *LIPUS* low-intensity pulsed ultrasound, *POI* primary ovarian insufficiency

To investigate the effects of hAD-MSCs and LUPUS-pretreated hAD-MSC transplantation on ovarian function in POI rats, the estrous cycle and serum levels of AMH, E2, and FSH were detected. The results show that 50% of rats had irregular estrous cycle in the first week after chemotherapy, and 100% of rats remained at diestrus from the third week after chemotherapy. At 1, 4, and 8 weeks after hAD-MSC transplantation, vaginal smears from the four groups were observed (Figs. 5a and b). The results show that the percentages of rats with abnormal cyclicity are decreased in both the hAD-MSCs and LIPUS + hAD-MSCs groups from the first week after cell transplantation.

On the other hand, compared to the POI group, the levels of AMH (indicating ovarian reserve) was significantly increased in the hAD-MSCs and LIPUS + hAD-MSCs groups, starting from the second week after cell transplantation (*P* < 0.01; Fig. 5c). Moreover, compared to the POI group, the levels of E2 were significantly increased in the hAD-MSCs and LIPUS + hAD-MSCs groups, starting from the eighth week after cell transplantation (Fig. 5d), while the FSH level was significantly decreased, starting from the second week after cell transplantation (Fig. 5e). However, there was no significant difference between the hAD-MSCs and LIPUS + hAD-MSCs groups. These results demonstrate that both hAD-MSCs and LIPUS-pretreated hAD-MSC transplantation can protect the ovarian reserve and improve the ovarian function in POI rats.

### Transplantation of hAD-MSCs reduces reproductive organ injuries in POI rats

**Fig. 6:**
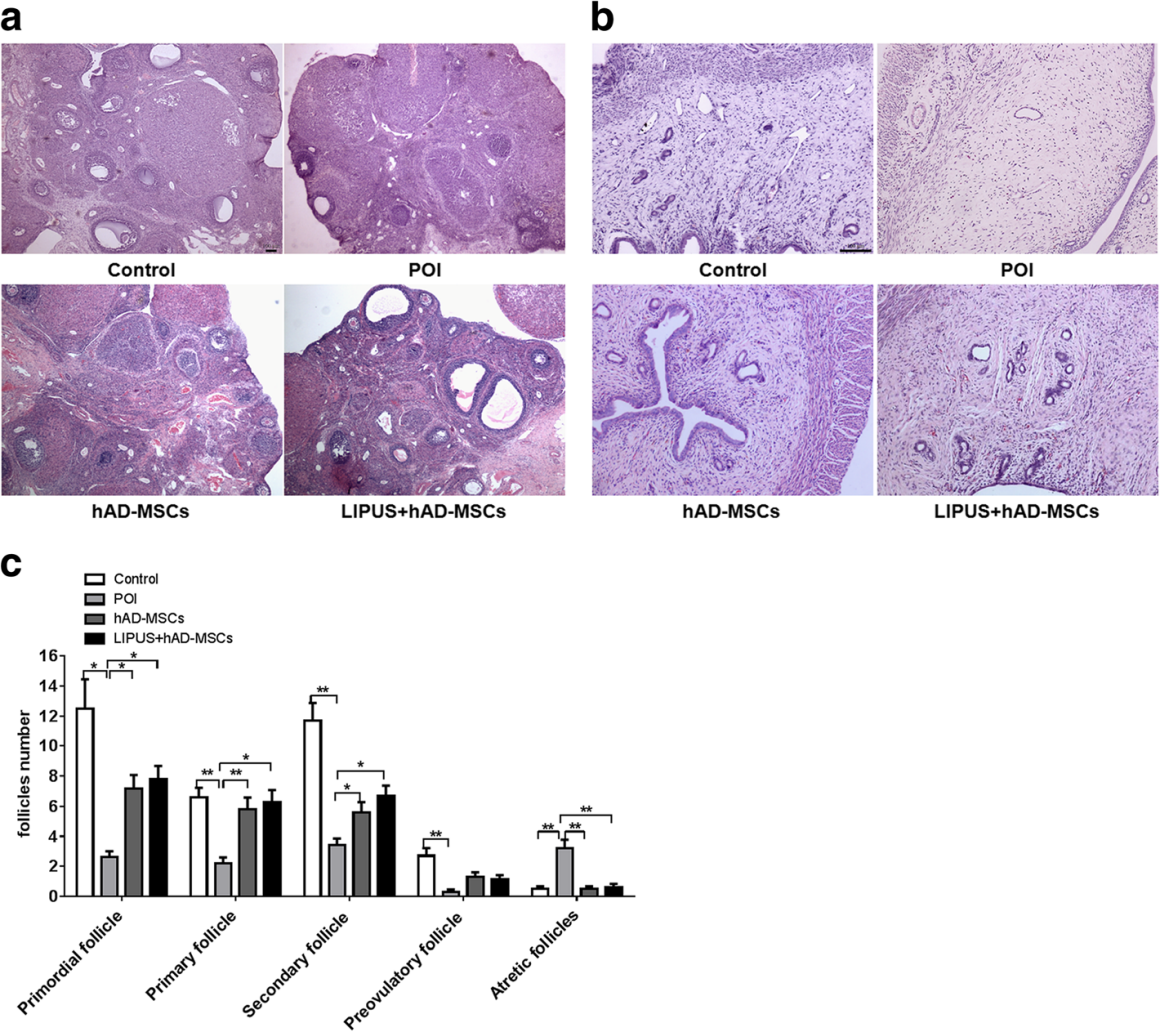
Transplantation of hAD-MSCs reduces reproductive organ injuries in POI rats. **a,b** The pathological changes of ovaries (**a**; 40×) and uteruses (**b**; 100×) were evaluated by H&E staining in the four groups at 1 month after hAD-MSC transplantation. **c** The number of follicles at various stages were counted and compared in the four groups. *Scale bars* = 100 μm. *P < 0.05, **P < 0.01. *hAD-MSCs* human adipose-derived mesenchymal stem cells, *LIPUS* low-intensity pulsed ultrasound, *POI* primary ovarian insufficiency

Ovaries and uteruses in the four groups were collected and subjected to pathological analysis. Our results show that there were significantly fewer primordial, primary, secondary, and preovulatory follicles in the POI group than in the control group, while there were significantly more atretic follicles in the POI group than in the control group (*P* < 0.05; Fig. 6a and c). Moreover, compared to the POI group, the number of follicles at various stages was significantly increased in both the hAD-MSCs and LIPUS + hAD-MSCs groups at 1 month after cell transplantation (*P* < 0.05; Fig. 6a and c), while the number of atretic follicles was significantly decreased in the hAD-MSCs and LIPUS + hAD-MSCs groups. However, there was no significant difference between the hAD-MSCs and LIPUS + hAD-MSCs groups. The main pathological change in the uterus in the POI group was the atrophy of uterine endometrium and myometrium due to a lack of estrogen (Fig. 6b). These changes were improved by cell transplantation in the hAD-MSCs and LIPUS + hAD-MSCs groups. However, there was no significant difference between these two groups. Graft-versus-host disease was not found in any transplanted rats. No obvious organic lesions were seen in the livers, hearts, lungs, spleens, or kidneys after chemotherapy. Taken together, these results demonstrate that both hAD-MSCs and LIPUS-pretreated hAD-MSC transplantation can reduce the injuries to reproductive organs in the POI rats

### Transplantation of hAD-MSCs reduces ovarian GC apoptosis in POI rats

**Fig. 7:**
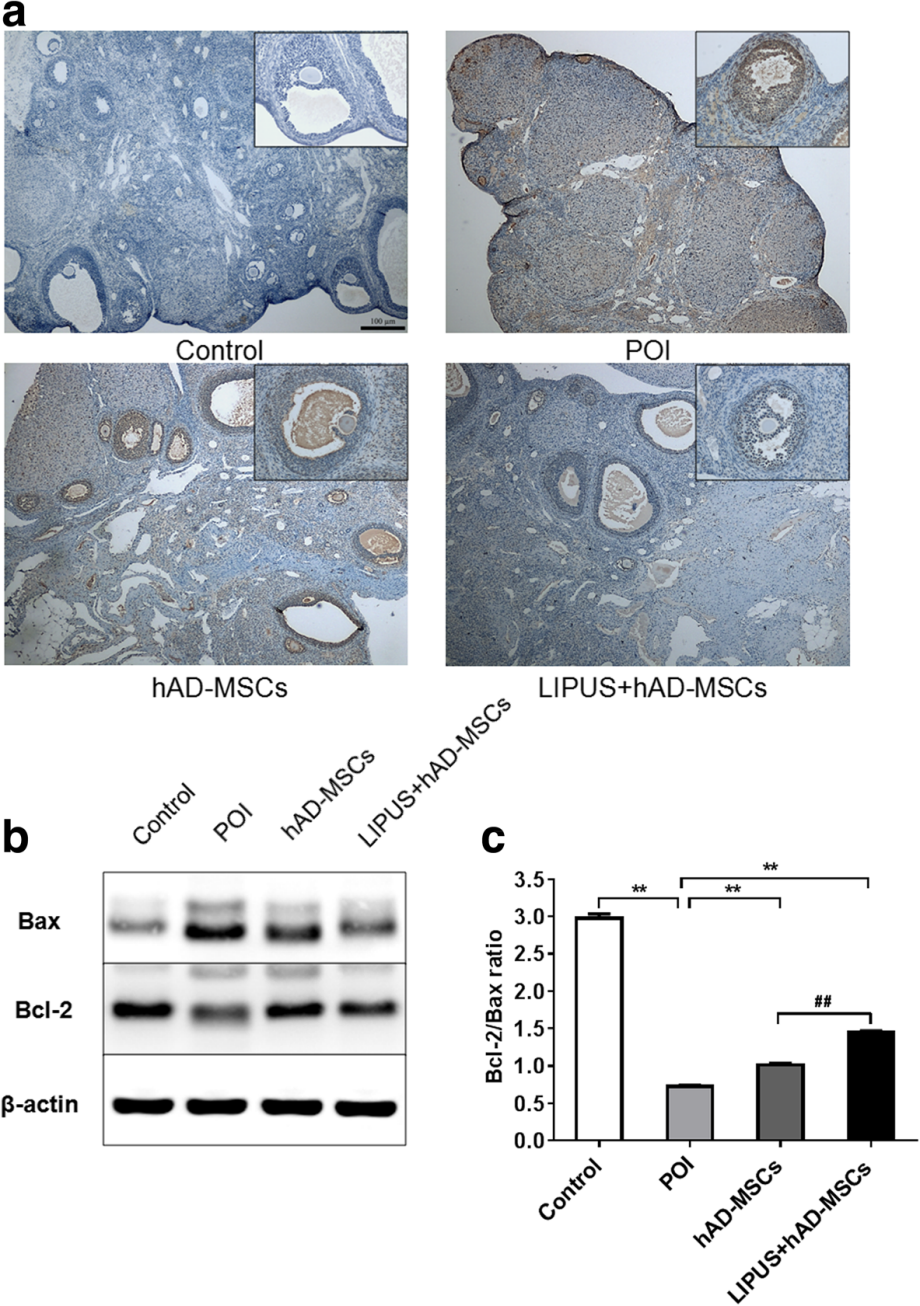
Transplantation of hAD-MSCs reduces ovarian CG apoptosis in POI rats. **a** Dark brown cells indicate the ovarian apoptotic cells stained by TUNEL apoptosis assay kit (40× and 200×). **b,c** Bax and Bcl-2 expression levels were determined by Western blot analysis (**b**), and the Bcl-2/Bax ratios were evaluated (**c**). *Scale bars* = 100 μm. *P < 0.05, **P < 0.01; ^##^ *P* < 0.01. *hAD-MSCs* human adipose-derived mesenchymal stem cells, *LIPUS* low-intensity pulsed ultrasound, *POI* primary ovarian insufficiency

To explore the effects of hAD-MSC transplantation on ovarian cell apoptosis induced by chemotherapy, TUNEL staining was used at 1 month after cell transplantation. Our results show that a large number of apoptotic GCs in ovaries were observed in the POI group. The number of apoptotic GCs in both the hAD-MSCs and LIPUS + hAD-MSCs groups was significantly less than in the POI group. Moreover, the number of apoptotic GCs in the LIPUS + hAD-MSCs group was less than in the hAD-MSCs group (Fig. 7a). The expression levels of apoptosis-related proteins, i.e., Bax and Bcl-2, in the ovaries were detected and quantified using Western blot analysis (Fig. 7b and c). Our results show that, compared to the control group, the levels of Bax were significantly increased (*P* < 0.01) while the levels of Bcl-2 were significantly decreased (*P* < 0.01) in the POI group. On the other hand, compared to the POI group, the levels of Bax were significantly decreased while the levels of Bcl-2 were significantly increased by cell transplantation in both the hAD-MSCs and LIPUS + hAD-MSCs groups (*P* < 0.01). The Bcl-2/Bax ratio in the POI group was significantly lower than in the other three groups (all *P* < 0.01). Compared to the POI group, the hAD-MSC transplantation significantly increased the Bcl-2/Bax ratio in the hAD-MSCs and LIPUS + hAD-MSCs groups (*P* < 0.01). Moreover, the Bcl-2/Bax ratio in the LIPUS + hAD-MSCs group was significantly higher than in the hAD-MSCs group (*P* < 0.01). These results demonstrate that hAD-MSCs can reduce the ovarian GC apoptosis and increase the Bcl-2/Bax ratio in POI rats, and the effect could be improved by pre-treatment with LIPUS.

### Transplantation of hAD-MSCs reduces ovarian inflammation induced by chemotherapy

**Fig. 8:**
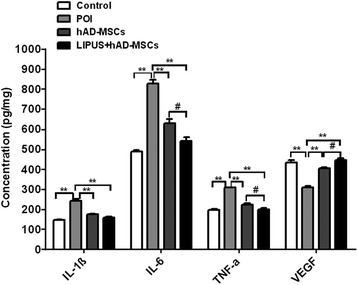
Transplantation of hAD-MSCs reduces ovary inflammation induced by chemotherapy. Expression levels of pro-inflammatory cytokines (IL-1β, IL-6, and TNF-α) and VEGF in the ovaries were detected by ELISA assay. * *P* < 0.05, ** *P* < 0.01; ^#^ *P* < 0.05. *hAD-MSCs* human adipose-derived mesenchymal stem cells, *IL* interleukin, *LIPUS* low-intensity pulsed ultrasound, *POI* primary ovarian insufficiency, *TNF* tumor necrosis factor, *VEGF* vascular endothelial growth factor

To determine the effect of hAD-MSC transplantation on ovarian inflammation induced by chemotherapy in POI rats, the levels of pro-inflammatory cytokines and VEGF in the ovaries were detected (Fig. 8). It was found that, compared to the control group, the levels of several pro-inflammatory cytokines (IL-1β, IL-6, and TNF-α) were significantly increased (*P* < 0.01) while the VEGF level was significantly decreased (*P* < 0.01) in the POI group. Compared to the POI group, the levels of pro-inflammatory cytokines were significantly decreased (*P* < 0.01) while the VEGF levels were significantly increased (*P* < 0.01) after hAD-MSC transplantation in both the hAD-MSCs and LIPUS + hAD-MSCs groups. Moreover, the levels of IL-6 and TNF-α in the LIPUS + hAD-MSCs group were significantly lower than in the hAD-MSCs group (*P* < 0.05), while the VEGF level in the LIPUS + hAD-MSCs group was significantly higher than in the hAD-MSCs group (*P* < 0.05). These results demonstrate that hAD-MSCs can reduce ovarian inflammation induced by chemotherapy and increase the expression of VEGF in ovarian tissue, and that this effect could be improved by pre-treatment with LIPUS.

## Discussion

In our study the results showed that hAD-MSCs isolated from the human amnion had the common characteristics of multipotent MSCs, and the procedure to obtain hAD-MSCs was noninvasive, safe, and out of ethical debate, consistent with previous studies [10–12]. In our experiments, a large number of hAD-MSCs can be isolated from human term amnion and expanded rapidly in vitro. The xenotransplantation of hAD-MSCs did not induce xenogeneic immune responses in rats with POI, suggesting the low immunogenicity of hAD-MSCs. These results demonstrate that hAD-MSCs may represent promising seed cells for tissue engineering and clinical applications in the near future.

Ovarian dynamics are characterized by repeated proliferation and differentiation of follicular cells, which is regulated by steroids, growth hormones, gonadotrophins, and a variety of intra-ovarian growth factors, including IGF, FGF2, VEGF, and HGF [39]. LIPUS is a form of mechanical vibration energy transmission. The acoustic pressure wave produced by LIPUS is able to transmit into and through living cells, which may result in a series of biochemical events at the cellular level [17, 18]. In contrast to high-intensity continuous ultrasound, LIPUS is a pulsed emission with low-intensity and low thermal effect, with minimal or no adverse effects on cells [40]. Several studies have found that LIPUS can enhance the production of VEGF in human mandibular osteoblasts, human peripheral blood monocytes, and human osteoblasts [41, 42]. Doan et al. [41] found that LIPUS treatment can upregulate the production of FGF2 in human mandibular osteoblasts. Moreover, Naruse et al. [43, 44] found that LIPUS can upregulate IGF-1 in rat osteoblasts and the murine ST2 bone marrow-derived cell line. These growth factors are crucial for angiogenesis and follicle growth in the ovary, which inhibits GC apoptosis [26–29]. In our experiments, our results showed that LIPUS could promote the gene and protein expression of IGF-1, HGF, and VEGF in hAD-MSCs, which suggested that transplantation of LIPUS-pretreated hAD-MSCs might be more advantageous to treat patients with POI.

In this study, the effects of hAD-MSCs and LIPUS-pretreated hAD-MSCs on chemotherapy-induced POI were explored. Our results showed that both hAD-MSC and LIPUS-pretreated hAD-MSC transplantation dramatically increased the body and reproductive organ weights of POI rats, and repaired ovarian injury and improved ovarian function. Although hAD-MSCs successfully recovered the ovarian function in rats with POI, they were only found in the interstitium of ovaries and did not differentiate into follicle components after transplantation. The results indicate that hAD-MSCs may restore ovarian function of chemotherapy-induced POI through paracrine effects, rather than through differentiating into GCs or oocytes. Previous studies have found that MSCs can inhibit cell apoptosis and enhance cell survival through paracrine pathways [14, 45], which thus exert positive effects on repairing ovarian tissue damage [8, 36]. It has been proven that human MSCs can secrete various cytokines, such as IL-6, IL-8, IL-10, IL-11, IL-15, VEGF, granulocyte colony-stimulating factor (G-CSF), HGF, IGF-1, and FGF2 [46, 47]. In our study, we focused on FGF2, IGF-1, HGF, and VEGF, which are likely to play important roles in restoring ovarian function in POI animals via stem cells [7, 46, 47]. It was found that hAD-MSCs can secrete FGF2, IGF-1, HGF, and VEGF, and the expression and secretion of IGF-1, HGF, and VEGF in hAD-MSCs could be promoted by LIPUS treatment. Our results further showed that, although both hAD-MSCs and LIPUS-pretreated hAD-MSCs could reduce inflammation, improve local microenvironment, and inhibit GC apoptosis induced by chemotherapy in ovarian tissue, the efficacy of LIPUS-pretreated hAD-MSCs were superior to hAD-MSCs. Thus, we speculate that the repaired ovarian tissue damage and improved ovarian function are more likely to be partially mediated by those growth factors produced by hAD-MSCs through the paracrine pathway.

As we know, scholars have verified the existence of ovarian germ stem cells, which can differentiate into reproductive cells and primitive GCs to replenish the follicle pool [48, 49]. The stem cell microenvironment, also called the stem cell niche, plays an important role in tissue repair after injury. The germ stem cell niche is composed of cytokines, extracellular matrix, and niche cells (mainly including GCs and vascular endothelial cells) surrounding the ovarian germ stem cells [50, 51]. Studies have showed that ovarian function failure is mainly due to the aging of the ovarian germ stem cell niches but not due to the aging of the ovarian germ stem cells [51–53]. Scholars have successfully separated ovarian germ stem cells from the ovaries of mice and humans with POI [51, 54], and ovarian germ stem cells from POI ovaries have the ability to differentiate into oocyte in vitro [54]. Thus, the microenvironment in ovarian tissue plays an important role in ovarian function failure and aging [51], and improving the microenvironment for ovaries of POI might make the ovarian germ stem cells remain active and continue to replenish the follicle pool. Winkler et al. found that CTX injured the stem cell niche [55]. Sun et al. found that transplantation of BM-MSCs was effective in inducing osteoblastic niche reconstruction and improving the hematopoietic stem cell niche [56]. Studies showed that CTX broke the inflammatory balance in ovaries through inhibiting the production of the anti-inflammatory cytokines and increasing the production of the pro-inflammatory cytokines [57, 58] and induced the apoptosis of GCs [25, 57], which is consistent with our results. Cytokines, GCs, and vascular endothelial cells are important components of the germ stem cell niche. Our studies found that hAD-MSC or LIPUS-pretreated hAD-MSC transplantation downregulated the expression of pro-inflammatory cytokines (IL-1β, IL-6, and TNF-α), which thus attenuated ovarian inflammation induced by CTX, upregulated VEGF, and reduced the ovarian GC apoptosis in the ovaries of POI rats. VEGF is an effective mitogen for vascular endothelial cells and a critical cytokine for angiogenesis [59]. Improving VEGF expression in the ovary is helpful for ovarian angiogenesis, GC survival, and follicle growth [60, 61]. Thus, hAD-MSC or LIPUS-pretreated hAD-MSC transplantation might improve the germ stem cell niche. Additionally, it is reported that MSC transplantation reduced the depletion of germ stem cells induced by CTX [4]. From these results, we speculate that hAD-MSC transplantation might reduce the depletion of ovarian germ stem cells and improve the germ stem cell niches through paracrine pathways, which might promote ovarian germ stem cells to differentiate into primordial follicles. On the other hand, Lai et al. also showed that MSC transplantation further reduced depletion of follicles at various stages after chemotherapy, including primordial follicles [4]. In this study, we found that the number of primordial follicles increased in both the hAD-MSCs and LIPUS + hAD-MSCs groups. Our results showed that the efficacy of LIPUS-pretreated hAD-MSCs was superior to hAD-MSCs in improving the ovarian microenvironment in rats with POI. The number of primordial follicles in the LIPUS + hAD-MSCs group increased more than in the hAD-MSCs group. However, there was no significant difference between these two groups. Further research is needed to investigate the mechanism underlying the effect of LIPUS + hAD-MSCs and hAD-MSCs on primordial follicles.

The POI rat model was established using CTX, which has been found to be able to cause POI in humans [25]. Moreover, CTX could induce apoptosis of GCs [25] which are required for oocyte survival and follicle development [62, 63]. In this study, our results found that GC apoptosis was significantly increased in the POI group as CTX was administered to the rats. After hAD-MSC or LIPUS-pretreated hAD-MSC transplantation, hAD-MSCs or LIPUS-pretreated hAD-MSCs dramatically reduced the ovarian GC apoptosis in the developing follicles, and the LIPUS-pretreated hAD-MSCs were found to be more advantageous in reducing GC apoptosis. Regulation of apoptosis is a very complex process, mainly involving two signaling transduction pathways: the extrinsic and intrinsic pathways. The extrinsic pathway starts at the cell surface by activating the cell death receptors, including the tumor necrosis factor receptor (TNFR). TNFR receives the death signal from TNF-α, which initiates a pathway that finally leads to apoptosis. In our study, our results showed that both hAD-MSCs and LIPUS-pretreated hAD-MSCs can downregulate the expression of TNF-α in POI ovaries, and the expression of TNF-α in the LIPUS + hAD-MSCs group was significantly lower than in the hAD-MSCs group. The results showed that hAD-MSCs may inhibit ovarian GC apoptosis partially through reducing the expression of TNF-α in ovaries, and this effect may be promoted by pre-treatment with LIPUS on hAD-MSCs. On the other hand, the intrinsic pathway starts from the mitochondria, which can be activated by chemotherapy. Activation of the intrinsic pathway is primarily regulated by the Bcl-2 family, among which the pro- and anti-apoptotic molecules (Bax and Bcl-2) are the most common markers for apoptosis [64]. Hussein et al. [65] found that one of the primary mechanisms involved in ovarian cell apoptosis is the intrinsic pathway, which is mediated by the BCl-2 family members. Bcl-2/Bax acts as a regulator in the follicular maturation and atresia. The Bcl-2/Bax ratio plays an important role in regulating ovarian cell apoptosis, which determines the fate of ovarian GCs [66]. Thus, we explored the expression of Bax and Bcl-2 proteins in our four groups. Our results showed that ovarian injury induced by chemotherapy resulted in increased Bax expression and decreased Bcl-2 expression, which reduced the Bcl-2/Bax ratio in POI rats. Both hAD-MSC and LIPUS-pretreated hAD-MSC transplantation significantly reduced the Bax expression and significantly increased the Bcl-2 expression and the Bcl-2/Bax ratio in the ovaries of POI rats. These results indicate that hAD-MSCs can protect ovarian cells against apoptosis by upregulating the Bcl-2/Bax ratio and that they play an anti-apoptotic role in POI.

Previous studies have shown that growth factors can induce the expression of anti-apoptotic Bcl-2 family members [67, 68] and reduce the expression of pro-apoptotic Bax [26]. In our study, LIPUS-pretreated hAD-MSCs showed the benefit of increased Bcl-2/Bax ratio and anti-apoptotic effects compared with the hAD-MSCs. Thus, we speculate that the mechanisms may be attributed to the increased growth factors in hAD-MSCs promoted by LIPUS, which increases the Bcl-2/Bax ratio; this needs further studies to confirm in the future.

## Conclusion

In conclusion, hAD-MSCs have the common characteristics of multipotent MSCs. Both hAD-MSC and LIPUS-pretreated hAD-MSC transplantation could repair ovarian injury and improve ovarian function in rats with chemotherapy-induced POI. LIPUS-pretreated hAD-MSC transplantation is more advantageous in reducing inflammation and inhibiting GC apoptosis induced by chemotherapy in the ovarian tissue. The efficacy of hAD-MSCs is more likely to be partially mediated by growth factors produced by hAD-MSCs through the paracrine pathway. These findings suggest that LIPUS may provide a way to promote the hAD-MSC secretome, and may be useful in MSC transplantation or a cell-free therapeutic approach (absence of stem cells) for the disease treatment in the clinic.

## Abbreviations

AMH: Anti-Müllerian hormone
ANOVA: Analysis of variance
BM-MSC: Bone marrow-derived mesenchymal stem cell
CCK-8: Cell counting kit-8
CTX: Cyclophosphamide
DAPI: 2-(4-amidinophenyl)-6-indolecarbamidine dihydrochloride
EDTA: Ethylenediaminetetraacetic acid
ELISA: Enzyme-linked immunosorbent assay
FDA: Food and Drug Administration
FGF: Fibroblast growth factor
FITC: Fluorescein isothiocyanate
FSH: Follicle-stimulating hormone
GC: Granulosa cell
H&E: Hematoxylin and eosin
hAD-MSC: Human amnion-derived mesenchymal stem cell
HGF: Hepatocyte growth factor
IGF: Insulin-like growth factor
IL: Interleukin
LIPUS: Low-intensity pulsed ultrasound
MSC: Mesenchymal stem cell
OCT: Optimal cutting temperature
PE: Phycoerythrin
POF: Premature ovarian failure
POI: Primary ovarian insufficiency
RT-qPCR: Real-time quantitative polymerase chain reaction
SD: Sprague-Dawley
TNF: Tumor necrosis factor
TNFR: Tumor necrosis factor receptor
VEGF: Vascular endothelial growth factor
